# Psychological empowerment after presbyopia correction: A narrative from Zanzibari women and men

**DOI:** 10.1016/j.ajoint.2025.100098

**Published:** 2025-04-28

**Authors:** Christine Graham, Omar Juma Othman, Adam Ali, Eden Mashayo, Ronnie Graham, Fatma Omar, Ving Fai Chan

**Affiliations:** a Queen's University Belfast Centre for Public Health, Belfast, UK; b Ministry of Health. Zanzibar, Mkoa wa Unguja Mjini Magh, Tanzania; c Ministry of Education and Vocational Training, Zanzibar. Tanzania; d Vision Care Foundation, Dar-es-Salaam, Tanzania; e Independent researcher. Edinburgh, UK

**Keywords:** Presbyopia, Women empowerment, Zanzibar, Physiological empowerment

## Abstract

**Purpose:**

To explore the empowerment experiences of Zanzibari craftswomen (*n* = 19) who received presbyopia correction, as well as perspectives from their husbands (*n* = 5) and male community leaders (*n* = 4).

**Design:**

Qualitative phenomenological approach

**Methods:**

Nineteen craftswomen who received presbyopia correction, five of their husbands and four community leaders were participated in the interviews. Craftswomen participants represented various crafts and locations across Zanzibar (Unguja *n* = 10, Pemba *n* = 9), with interviews conducted in Swahili. Data collection involved semi-structured interviews, and analysis employed both inductive and deductive thematic techniques.

**Results:**

Craftswomen reported that wearing spectacles enhanced their ability to work, increased productivity, and improved confidence and independence. Psychological empowerment (PE) was evident in their decision-making and autonomy, as well as their capacity to support their families financially. Men observed the positive effects of spectacles on the women's self-reliance and community contributions. Participants noted that community attitudes toward wearing glasses were generally supportive, although some stigma persisted.

**Conclusion:**

Presbyopia correction through spectacles facilitated significant PE among Zanzibari women, enhancing their independence, confidence, and productivity. A supportive community environment further bolstered empowerment, underscoring the importance of accessible eye care in improving quality of life and well-being.

**Precis:**

We explored the empowerment experiences of 19 Zanzibari craftswomen following presbyopia correction, incorporating perspectives from their husbands and community leaders. Using a qualitative phenomenological approach, semi-structured interviews revealed enhanced productivity, independence, confidence, and psychological empowerment among the women. Male participants observed increased self-reliance and community contributions. While community attitudes were supportive, some stigma persisted. This highlights the transformative impact of accessible eye care on women's quality of life and underscore its broader societal benefits.

## Highlights

• This is the first study to have used qualitative phenomenological method to understand psychological empowerment through correcting presbyopia of older women from the perspective of both women and men.
• Wearing glasses empowered women by improving their independence, productivity, and decision-making abilities in both personal and professional contexts.
• Women reported increased income and financial empowerment as a result of improved work efficiency while they were able to contribute to household expenses, save money, and make independent financial decisions, with similar observations noted by men in the community.
• Improved vision increased their ability to participate in social and political activities, and men observed this empowerment as beneficial to family and community dynamics.
• Men recognized the importance of empowering women and believed that fostering women's autonomy, access to education, and economic participation would positively impact society, demonstrating a community-wide commitment to women's empowerment.

## 1 Introduction

The 1978 Alma Ata Declaration recognized health, including eye care, as a human right essential for quality healthcare.^1^ However, eye care still faces challenges in availability, accessibility, affordability, acceptability and sustainability,^2^ leading to preventable blindness and vision impairment that negatively impact quality of life and equitable access to resources.^3^

Approximately 2.2 billion people have vision impairment, with over 1 billion cases preventable through timely access to quality eye care.^2^ Uncorrected presbyopia affects 510 million people globally, over half of whom are women,^4^ with most cases concentrated in low- and middle-income countries (LMICs).^3^ Vision impairment, particularly presbyopia, is common in older populations,^2^ with women at greater risk due to longer life expectancy and socio-economic barriers to eye care.^3^^,^^5^ Factors such as limited education, inadequate healthcare access, and lack of social support impact health and increase isolation,^6^, ^7^, ^8^ and lower socio-economic status,^9^ contributing to poor psychological well-being. Gender disparities further disadvantage women in obtaining spectacles, heightening their risk of uncorrected presbyopia.^10^

In Zanzibar, gender inequalities have been targeted by policies promoting women's empowerment across all areas of life.^11^ As a semi-autonomous state of Tanzania with a population of 1.3 million (52 % women),^11^ a study found that 89.2 % of those aged 40 and over have presbyopia,^12^ yet only 17.6 % have spectacles.^12^ Key barriers include cost and lack of awareness, disproportionately affecting women,^12^ who are often without formal education or employment.^13^ This increases the economic burden on female-headed households.^11^ Providing spectacles could enhance women's social and psychological empowerment (PE).

Empowerment in global health aligns with the Ottawa Charter's concept of health promotion, defined as “*enabling people to control and improve their health*”.^14^ This involves control over health determinants in social, economic, political, and environmental contexts.^15^ PE plays a key role with Conger and Kanungo^16^ viewing it as a motivational construct that enhances self-efficacy, while Mas and Velhouse^17^ emphasized PE as a boost in task motivation and satisfaction through meaningful, positive experiences. Basically, PE is an ‘*internal incentive process, a continuous working motivation and mental state or a synthesis of cognition of being empowered’*^18^ and it is driven by four key perceptions: Meaningful work, competence, self-determination and impact.^17^, ^18^, ^19^, ^20^, ^21^ These contribute to a sense of personal control and influence over decision-making.^22^, ^23^, ^24^ Correcting presbyopia could thus empower individuals, enabling effective engagement in daily tasks and work.^25^

Few studies examined the impact of corrected presbyopia on PE. Our previous research found that women in Zanzibar saw presbyopia correction as enhancing personal and relational empowerment, boosting confidence, and supporting decision-making.^26^ Other studies in LMICs^2^^,^^3^ show that providing spectacles can improve well-being, especially socially and economically, by removing barriers like cost, access, and acceptance of services. Corrected presbyopia has been linked to improved quality of life^12^^,^^27^^,^^28^ and productivity,^29^ with gains of 6.4 % among textile workers in South Africa^30^ and 22 % among tea pickers in India.^31^ These findings show spectacles can maintain or improve the functioning and independence of individuals to enhance overall wellbeing^10^^,^^32^ and underscore the need for affordable, accessible eye care. The World Health Organisation recommends addressing vision impairment to empower individuals and communities.^2^

This study is a six month follow up of women empowerment after presbyopia correction. In this paper, we attempt to delve into PE as we examine Zanzibar women's experiences and other individuals from the community living close to these women. We will throw a light on how these experiences and observations of participants demonstrate meaningfulness, competence, self-determination, impact and trust in contributing to PE as vital to overall health and wellbeing.

## 2 Methodology

### 2.1 Study design

This is a qualitative study and adopts a phenomenological approach. Semi-structured interviews were used for data collection. We minimised researcher and respondent biases using experienced interviewers who were skilled and knowledgeable about the studied community. They were guided by the interview protocol.^33^ Additionally, set within a comfortable ambience, participants shared their daily experiences of wearing spectacles for the period of six months.

This study was part of a larger project, where 209 craftswomen 40 years and older participated. The participants were from Unguja and Pemba Islands, mostly aged 40–50 and had worked in their craft for 10 years or less.^34^ The prevalence of presbyopia in the population was estimated to be 86.7 % with only 2 person adequately corrected.^35^ A study of craftswomen's perception on vision and empowerment (before correction) revealed that they perceived that correcting near vision impairment could lead to various forms of empowerment, including economic, social, psychological, educational, and political benefits.^26^ The current study is the qualitative component of the project to understand the PE among the craftswomen six months after the correction.

### 2.2 Participants

As this was a follow-up study, it included craftswomen who had received eyeglasses in an earlier study. Participants were selected to represent a variety of crafts— weaving (*n* = 5), tailoring and sewing (*n* = 5), pottery (*n* = 5), and oil production and soap-making (*n* = 4)—as well as different locations (Unguja *n* = 10, Pemba *n* = 9) and experience levels (<10 years, *n* = 11; >10 years, *n* = 8). This selection ensured a rich understanding of the women's experiences with vision correction and PE. The sample size aimed for data saturation, prioritizing depth and diversity of experiences rather than statistical representation. Additionally, we included five husbands of the craftswomen and four male community leaders. Including men in discussions promotes broader community support and understanding of vision correction benefits, enhancing acceptance and empowering women through shared perspectives.

### 2.3 Data collection

Semi-structured interviews were used for this study. Two interviewers (FO, Female, MSc; OO, Male, MA) conducted and recorded the interviews in Swahili to ensure accuracy. We pursued Lincoln and Guba's ‘trustworthiness’ framework to strengthen the quality of the study.^36^ The framework standards include credibility - the truth of the data; dependability - the stability of data and authenticity - demonstrating accurately the realities of the study group. To establish credibility and authenticity, audio recordings were transcribed in Swahili (AA, Male, BA), translated into English (AA) and then back-translated by a public health specialist and a Swahili-speaking analyst (CG, Female, PhD). With regard to dependability, we followed up any ambiguous responses with participants. Finally, an independent reviewer checked the transcripts against the audio to corroborate the quality of data.

### 2.4 Data analysis

A phenomenological approach was used for analysis where the emphasis was anchored on meanings from the experiences of the study participants.^37^ The data obtained was analysed using inductive thematic analysis.^33^ The analysis process started with two analysts (CG; VFC, Male, PhD) reading the transcripts to familiarise themselves with the data.^38^ The transcripts were then manually coded using Microsoft Word®, then condensed meaning units were assigned to the codes, thereby informing us of the emerging themes. Each participant was labelled according to the crafts cooperative they belonged to and given an assigned number. Throughout this process the analysts were first independently coding each transcript then discussing the codes and meaning units. The themes were then determined. Since the empowerment concept is a multi- level construct where levels of analysis are interdependent,^22^ we also applied deductive analysis to get a better understanding of the women's narratives. We adopted psychological perspectives by pursuing ‘*an individually oriented conception of empowerment*’ for our analysis.^23^ These include **impact** – the opportunity to make choices and the apparent effect of one's work on outcomes; **meaningful life/work** – to engage in activities and being fulfilled and evaluate work according to own values; **competence/self-efficacy** – to be confident in their capacity to make choices; **self-determination –** to be independent, make their own decisions and have control over actions; **trust** – the creation of atmosphere for empowerment growth.^6^^,^^18^^,^^20^^,^^21^^,^^23^ We used these concepts as sub-themes to elaborate further meaning in the women's narratives.

### 2.5 Ethical issues

The study was approved by the Ethics Committees from the Zanzibar Human Research Institute (ZAHREC/04/PR/MARCH/2022/12), Zanzibar Office of the Government Chief Statistician (6221C2601263D) and Queen's University Belfast (MHLS 22_72). Informed consent was obtained from all participants, confidentiality was assured and participation was entirely voluntary.

## 3 Results

### 3.1 Theme 1: the realities of wearing glasses

Women's experiences with wearing glasses were diverse, encompassing both positive and negative aspects. Some women (*n* = 8) initially faced challenges, such as discomfort, headaches, and stigma from the community [Quotes 1–6]. However, most participants (*n* = 16) described wearing glasses as transformative, enabling them to regain control over their vision and daily lives. This transformation was particularly evident in their ability to engage in professional and personal tasks with greater ease [Quotes 7, 8]

Women's feelings about these experiences gave an impression of empowerment which could be psychologically categorised in terms of the choice of wearing glasses has **impacted on** their daily lives and work; how they evaluate their work as **meaningfu**l; their **competenc**e in embracing the benefits of wearing of glasses which increased their self-confidence and **self-determination,** where women felt a sense of independence and the ability to make decisions about their work and income.

#### 3.1.1 Subtheme 1: impact

Wearing glasses significantly improved women's near vision, allowing them to engage in crafts like sewing, knitting, and weaving more effectively [Quotes 9-12]. Others noted enhanced ability to read and handle paperwork, which was empowering and crucial for managing household and business-related tasks [Quotes 13, 14]. Men confirmed these observations, reporting that improved vision reduced women's reliance on others, particularly children, for assistance with daily activities [Quotes 15–17].

Women also highlighted their increased productivity, attributing it to better vision. They expressed satisfaction with their ability to work faster and produce higher-quality goods, which was reflected in their increased income and sense of accomplishment.

#### 3.1.2 Subtheme 2: self-determination

The use of glasses fostered a profound sense of independence among women. They felt more in control of their lives and capable of making decisions independently, particularly in financial matters. This newfound autonomy was reinforced by increased income, allowing women to save, contribute to household expenses, and support their families without external assistance [Quotes 18–33].

Men recognized these changes and noted how women's financial independence positively impacted the family's overall well-being. They observed that women's ability to manage resources effectively contributed to household stability and reduced financial strain [Quotes 34–39].

#### 3.1.3 Subtheme 3: competence/self-efficacy

Improved vision bolstered women's confidence and competence, enabling them to perform tasks with greater proficiency and ease. Participants reported feeling empowered to support their families, participate in social and political activities, and take on leadership roles in their communities [Quotes 40–43].

Men echoed these sentiments, emphasizing how glasses equipped women with the tools to overcome barriers and engage actively in community affairs. They noted that improved self-efficacy fostered a sense of pride and accomplishment among women [Quotes 44–48].

#### 3.1.4 Subtheme 4: meaningful work

Women's work became more meaningful after vision correction. They expressed satisfaction with their ability to work for longer hours and produce goods of higher quality. These improvements were not only fulfilling but also translated into tangible economic benefits, such as increased income and greater contributions to household finances [Quotes 49–53].

Men agreed, noting that women's enhanced productivity elevated their role in the family and community. They observed that the ability to work more efficiently and independently strengthened women's sense of purpose and value [Quotes 54–56].

### 3.2 Theme 2: community behaviour towards women/girls wearing glasses and women's empowerment

Community attitudes toward women wearing glasses reflected mixed behaviors. While some women (*n* = 5) experienced stigma and social disapproval, many reported that wearing glasses was increasingly normalized within their communities. Trust emerged as a critical factor for empowerment, with participants emphasizing the need for a supportive environment that fosters collaboration and autonomy.

#### 3.2.1 Subtheme 1: entrenched behaviours within the community

Stigmatisation was a pattern of behaviour highlighted by a few women. To a certain extent, women felt socially disapproved of by their community while wearing glasses. In the larger community, stigma was claimed to happen with other eye health conditions as well [Quotes 57 and 58]. However, several women (*n* = 7) stated that to a greater extent individuals wearing glasses were being treated as normal in the community [Quotes 59 and 60]. Similarly, the majority of the male participants (*n* = 7) said that in the larger community, stigmatisation could rarely be observed [Quotes 61 and 62].

It is apparent that these descriptions of community conduct towards individuals wearing glasses have mixed characteristics. Mostly these individuals are considered as part of the community and in several instances, they are viewed indifferently, and it seems unlikely for women empowerment to strengthen where there are issues of fear and mistrust in the community. Accordingly, we analysed both women and men's opinions about the community setting, raising the concept of trust as a sub-theme.

#### 3.2.2 Subtheme 2: trust

Empowerment was closely linked to trust within the community. Participants emphasized the importance of a supportive environment where individuals trusted each other and collaborated to foster women's autonomy. Women and men highlighted the need for education, access to resources, and societal support as essential components of empowerment [Quotes 63–84].

Men recognized their role in this process, acknowledging that empowering women required shared responsibility and collective action. They stressed the significance of providing women with tools, opportunities, and the freedom to make independent decisions, which would ultimately benefit both families and the wider community [Quotes 85-87].

**Fig. 1:**
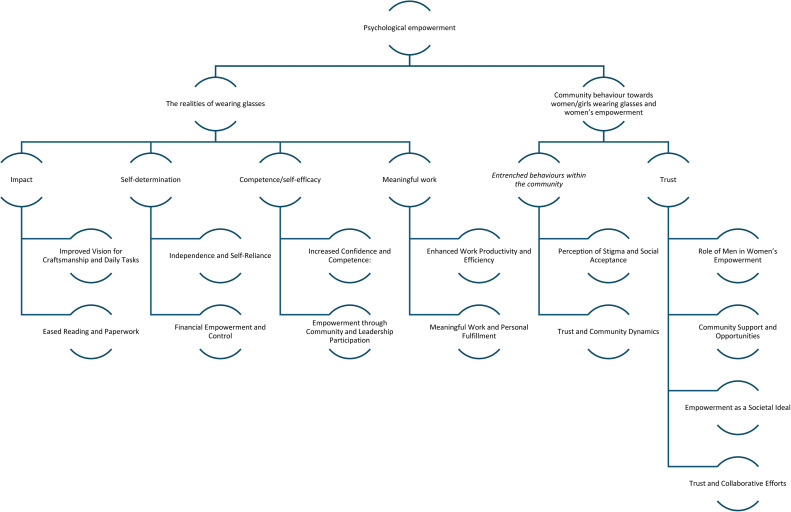
Thematic Map of Themes and Subthemes.

All quote excerpts are shown in Table 1 and the thematic mapping of key themes and subthemes are shown in Fig. 1.

**Table 1:** Quote excerpts from participants.

Quote number	Quote excerpts	Participant
1	‘I can see clearly when sewing dresses but not when I gaze far away.’	MM-3
2	‘I also have a problem … my major issue has been my inability to see far. I can see here and there, but not far.’	MW-2
3	‘My head hurts in the middle, which is the only negative experience I've had, so I can see that wearing glasses still presents challenges for me, especially if I wear them for an extended period of time.’	MW-1
4	‘Often I get a headache when I take off my glasses and my eyes hurt.’	CW-2
5	‘When we wear glasses, others are looking at us as we act like queen, they … call us four-eyed, but they do not know that we have problems. Everyone has their own interpretation because we are not all the same, you will see yourself wearing glasses with problems, but your partner will see you wearing them for show.’	MW-1
6	‘From my point of view, people think that we are showing off and pretending, but they don't know that we have eye problems, they see us too self-conscious.’	CW-1
7	‘I have not experienced anything bad or any irritation or any difficulties from using glasses.’	NYM-1
8	‘I have not experienced any bad experience.’	NT-3
9	‘… but now I'm thankful that when things are close, I can see clearly.’	NT-1
10	‘Apart from the difficulties I have gone through, the eyeglasses do help in some way because, it has improved my vision.’	MW-1
11	‘Since I wear glasses, I can sew, weave, and thread a needle with ease’.	NT-2
12	‘… at the moment, we can do all our work … in knitting, light work in sewing and various other jobs in our group and I have not had any bad experience since we got the glasses. We are really very relieved from the burden we had.’	MW-4
13	‘After I started wearing glasses, I am grateful that they have helped me because I was unable to read before. …I have been able to read well at work.’	NT-3
14	‘It has certainly improved us because we can see clearly and have the ability to fill in forms and read.’	NYM-1
15	‘For my part, I see that she has more and more of good vision, for example, she is now capable of putting strings into the needle, and do her best in sewing.’	One-2
16	‘For my fellow partner, it has helped her a lot because now she can read without squinting a bit.’	Two-2
17	‘It has also removed the inconvenience for the children because the children were already running away from their mother because they were called frequently to help put the needle on the thread.’	Two-3
18	‘My confidence has increased, … I have reduced dependence on a number of issues. On the other hand, I am not a person who is getting involved on the issues of leadership. It is enough for me to be a leader of my children.’	MW-1
19	‘I also see the meaning of wearing glasses because in the past I used to put two needles and call the child to put the string, but now I do it my myself.	MM-2
20	‘[Although] I had confidence, … the fact I get something to earn, has reduced dependence on my husband. This creates confidence on deciding my own affairs.’	MM-3
21	‘I have increased … earnings as well as saving, right now I have taken some of the responsibilities just to help my husband [such as] … buying things for the children and home cooking materials.’	CW-3
22	‘At least now we get a little to save and use for buying things for our children.’	NYM-3
23	‘I have the ability to make decisions about money.’	MW-4
24	‘Our earning now is different from the beginning, it has improved, not to the great extent, but the changes of income can be seen vividly.’	MW-1
25	‘Nowadays my earning increase gradually.’	MM-3
26	‘I am able to decide how to spend the money I make.’	NT-2
27	‘We have confidence that is built after the income increases and having support in the family.’	NYM-1
28	‘I can support my family since I earn a nice living.’	NT-2
29	‘I now make something to support my family and pay a little for my children's education.’	MM-4
30	‘I also have the ability to contribute to the household … and also buy the children even small things and run the family well.’	MW-4
31	‘In fact, now our income has increased a lot because initially we did not have any income due to our work performance, but it has increased.’	MW-3
32	‘We can pay the costs of the products and provide the equipment to make the products, and we got money and we shared it with the members of our group and we also have a CRDB [Co-operative Rural Development Bank] loan.’	NYM-4
33	‘Our confidence has increased; we can now take decision about many of our plans since those plans needs money that now can be generated from our own activities.’	MM-2
34	‘She is now capable of getting something to earn and save. Right now they have their decision of what to do with their money, we are only informed.’	One-2
35	‘I have not experienced any negative changes, with respect to income, it has increased somehow. She no longer tells me to give her money for buying small things of home cooking. She can manage herself.’	Two-1
36	‘To me, I can see positive changes is what we have and we continue to witness them, which includes increasing their confidence, their earning and saving.’	Three-3
37	‘… and I am really happy with the result because now she uses the little she gets to buy things for children.’	One-2
38	‘The difference is that we now find ourselves assisting each other with the responsibilities of raising children.’	Three-2
39	‘… the woman's money is not always visible, but they can contribute to the household. First of all, it is difficult to explain to you about her income, but she only helps the children well.’	Three-3
40	‘I am feeling very confident right now since we can perform our routine tasks, such as reading pamphlets, reading children's letters, and accepting orders from customers.’	MM-1
41	‘I am confident to decide my own affairs, but I have not decided yet to participate in the leadership.’	CW-1
42	‘…For me right now I value myself very much and I believe in myself very much and these glasses have helped us a lot and I can socialize with family or society.’	MW-2
43	‘I have the ability to decide in my household and help each other in anything. I can appear in public without any problem and be able to decide anything and also if I am elected to be a leader, I have confidence that I will be able to perform better.’	MW-4
44	‘For me, it also helps me a lot to decide my own affairs and I can participate in the leadership, in choosing someone, and participate in various things in the community, the glasses help me to do something in a short time’	CW-4
45	‘I used to run for office and I got the secretariat of CCM members and I am in the leadership of the secretariat. We are very confident in making contributions to the community and making various decisions and we also participate in events such as participating in the census exercise.’	NT-2
46	‘About confidence, it has increased since she can manage doing her work with little dependency.’	Two-1
47	‘They also have good self-confidence and can encourage their peers to join groups, and they have time to deal with the family and home.’	Three-1
48	‘To me, I can positive changes is what we have and we continue to witness them, which include increasing their confidence, … and some of them are expected to compete for political position once the time [reap up].’	Three-3
49	‘Due to more orders I have from different peoples [customers], … I don't have free time, the time I have is for completion of the order from the peoples [customer].’	MW-2
50	‘The rise [workload] is due, … to the fact that each member of our group received a tender for weaving four bags. …I now have good vision, I am able to work day and night …’	MM-1
51	‘Now I'm doing well and work is going well, before I got glasses, our work didn't go well. For example, [when] I want to thread a needle, it was difficult to do it, the work that takes five minutes, it took me a quarter of an hour to be completed. Now I am doing great with my eyeglasses.’	NYM-2
52	‘The reality for me is that my income has increased because I produce products in large quantities and in a short period of time and the products are of high quality.’	NYM-1
53	‘For me, I have gained a good experience by doing my work quickly and easily …’	MW-3
54	‘Their (women) handiwork goes well compared to the conditions they had from the beginning.’	One-1
55	‘I am fortunate to see, because I have seen that it has helped in her activities, now she has been quick in doing her work’	Two-1
56	‘What I discovered is that her work efficiency has increased, and thus her output has increased..’	Three-3
57	‘To my view, people have different views and they treat us differently. If you wear glasses and you are aged, they take it as normal, but if a younger person wears glasses, they take it as a show [pretentious].’	MW-2
58	‘[Stigma] exists because society does not prioritize the needs of those who are blind.’	MM-2
59	‘In the family, we have people who wear glasses, they … fully … participate in any family or society events, so there is not any bad treatment.’	NT-4
60	‘To my side I have not confronted any difficulties to my family or society due to short sightedness.’	NYM-2
61	‘Wearing glasses does not cause problems, and it is not for beauty. They are treated as normal people, … [we are always told] that these glasses are for eye therapy.’	Three-1
62	‘We have not noticed difference in treatment. I would like to make one thing clear, if a woman [cannot see properly], she will not be given work that require high vision capacity and that is not discrimination. We cannot say that, she has been treated differently.’	Two-1
63	‘We always need men, to support us doing anything for the benefit of our families and society,… they are the final decision maker in our societies.’	NT-3
64	‘Men have this role as they are leaders of our families as well as societies.’	MM-2
65	‘Men and the society should help empowering us in production of goods, finding the market, as well as in our future planning.’	MM-2
66	‘Let us take an example right now, we do feel in our group that if there are males, they could help us greatly by advertising our products, because they are usually roaming here and there.’	MW-4
67	‘It's very true that we should be helped by men and the society in general, …there are situations where it becomes difficult to handle as a woman, like looking for the market or transporting raw materials for our production. Men are in good position to assist us on that.’	NYM-2
68	‘We should be helped because women … usually … lack self-confidence or [feel] inferiority. …Men should be given education to support us because some men cannot support their wives in these groups. There are few women who can come forward, [without] the permission of the Lord (husband).’	NYM-1
69	‘Motivation (from men) to us is very important since [business] is not … good at all times. This will help us to focus on the elimination or reducing of the challenges encountered in our activities.’	NYM-3
70	‘They (men) should help us, for example, home affairs, and taking care of our health matters too.’	MW-1
71	‘There are a lot of areas that men can help to empower us. One of the areas is giving us permission to participate in production activities in our groups.’	NYM-3
72	‘We need to be empowered by both men and the society. For example, to be given projects of any kind so that we can move forward in life and not just going backwards every day.’	MW-1
73	‘Helping women is the same as helping the entire society. … In today's world, we see women as capable of great things; it is our responsibility to empower them because what they gain will be passed down to our children.’	Three-3
74	‘There are women who, when empowered politically, can be of tremendous assistance to the family and the community while distinguishing her power from her husband's position and her responsibilities to her husband and family.’	One-1
75	‘In my opinion, … it is giving her power that can help her manage the family, especially at a time that you are not there at home.’	One-1
76	‘A large percentage of women do not agree to be decided by their partners, that is, they often decide to empower themselves.’	Three-3
77	‘Women need to be … given education so that they are empowered, and they appear in large numbers in various government activities, this was [even] … before getting eyeglasses.’	Three-1
78	‘My thoughts are to empower her in a way that will enable her to get anything in her interest, to develop herself in life, or to empower her educationally or in life in general.’	One-2
79	‘[Empowered women] … groups have progressed and produced products in quantity according to the previous production and the profits have been many, which leads to an increase in income.’	Three-1
80	‘We usually empower women so that we can get benefits from them, including raising themselves in their entrepreneurial work to increase capital and profits that will lead to reducing family poverty.’	Two-1
81	‘Empowering [a woman] by giving her something that will help her in all the movements of her life, we aim at the end of the day for her condition to improve.’	One-3
82	‘Positive aspects of women empowerment include [attributes] that will change a woman in her work performance in the field of increasing her efficiency, being able to reduce dependence, increase confidence and other things.’	Two-3
83	‘Empowering a woman by giving her the chance to create a way for herself to meet her needs and those of her family.’	Three-3
84	‘Yes, that is our culture here in Zanzibar that it is our traditions and customs, even in religion, helping and giving are encouraged and it is one of the remedies to empower them firstly by providing education and secondly by providing them with resources or materials to develop that life and to lay down structures and methods for them by going to do small trades.’	One-1
85	‘For my part, empowering a woman means providing the capital she needs, for example if she is a seamstress, provide her with sewing tools or agricultural tools if she is a farmer or fishing tools if she is involving in fishing.’	Two-1
86	‘On my part, even our permission can empower our women, it is not a small thing but also giving them capital to start working on any type of entrepreneurship.’	One-2
87	‘Empowerment of women is the condition of a woman being given support either financially, intellectually or even helping her to make her work easier to succeed, such as finding markets for her.’	Two-3

## 4 Discussions

This study focused on women's PE following presbyopia correction, identified two main themes. The first, "realities of wearing glasses," highlights experiences aligned with PE concepts: impact, self-determination, competence, and meaningfulness. The second, "community attitudes towards women wearing glasses and empowerment," explores how entrenched community behaviors and trust-building contribute to women's empowerment.

Overall, vision impairment is associated with lower quality of life, whereby difficulty in performing visual tasks such as seeing and reading affects every day functioning.^28^ To date, there are no published studies from LMICs that qualitatively examined PE after presbyopia correction. Vision impairment is inevitable the older one becomes, where it could become a challenge in terms of motivation, resulting in reduced participation in certain social, economic and political opportunities.^9^^,^^39^ Women in this study reported that wearing glasses allowed them to see clearly, simplifying their lives both at home and in work environments. They validated their experiences, emphasizing how choosing to wear glasses positively influenced their psychological empowerment through self-determination. These findings align with studies showing that motivated, need-driven decisions can significantly enhance well-being.^9^ Comparable research in Durban found improved quality of life among textile workers following presbyopia correction.^40^ Conversely, studies reveal that individuals with uncorrected presbyopia face lower quality of life due to challenges in daily living and work.^39^^,^^41^ Our findings suggest that PE is rooted in the awareness of being in control and taking responsibility for outcomes, driven by self-determination.^20^

Notably, women reported a sense of PE after taking actions and making decisions that were not possible before presbyopia correction. This reflects their perception of self-determination, which emphasizes individual motivation to act autonomously—whether driven by intrinsic satisfaction or external pressures.^42^^,^^43^ In this study, women identified themselves as the source of their intrinsically motivated behaviors,^17^^,^^42^^,^^43^ which contributed to their feeling of empowerment. They described newfound autonomy, allowing them to function independently and take control of decision-making, particularly in social and financial contexts. By contrast, a study in Nigeria found that uncorrected presbyopia resulted in three times the dependency rate compared to those without presbyopia.^41^ After receiving correction, women in this study reported wearing glasses daily, enabling them to make meaningful changes in their everyday environments.^20^ Their self-determination in making these choices appeared to regulate behaviors aimed at fulfilling basic physical and psychological needs, which in turn could enhance their sense of competence.^44^

Findings from this study highlight increased competence and self-efficacy as key outcomes of presbyopia correction for women. Presbyopia often results in a decline in physical well-being, linked to psychological challenges such as social isolation caused by vision difficulties in daily environments.^45^ Despite these challenges, women in this study chose to wear glasses, enabling them to perform tasks proficiently and achieve their goals.^19^ This experience fostered competence, confidence, and the ability to make effective decisions across household, community, and workplace settings. Self-efficacy further reinforced their commitment to daily activities. However, competence and confidence alone are insufficient without access to resources or knowledge of how to obtain them.^24^ A lack of resources to address vision impairment may lead to dependency and a sense of hopelessness, undermining PE and everyday competence.^9^ Moreover, this dependency may erode individuals' sense of purpose or meaning in life.

Meaning in life, in the present study, means a quality of daily existence and promoting psychological and physical health.^46^, ^47^, ^48^ Women's prioritization of wearing glasses allowed them to function normally and achieve their goals, fostering a meaningful life.^47^ This was evident in their adherence to wearing glasses, which supported meaningful engagement in daily crafts. Meaningful work, known to enhance motivation, performance, and commitment, led to positive psychological outcomes such as contentment and well-being.^49^ The study revealed that women experienced greater efficiency, longer working hours, and increased productivity, which were previously unattainable. These findings align with studies in South Africa and India, where presbyopia correction improved productivity among textile workers and tea pickers, respectively.^30^^,^^31^ Meaning in life is further associated with positive health assessments, though healthier individuals may report higher meaning due to more active and less burdensome lives.^50^ However, the experience of meaning is usually located and realised in the mechanisms of mutual relations with others.^47^

This study highlights that women's experiences of empowerment were deeply rooted in their roles within households, craftswomen groups, and the broader community. Friendly and supportive environments were key to fostering empowerment.^23^ While wearing glasses was generally accepted, some participants experienced social disapproval, reflecting lingering stigma that has been linked to reduced use of presbyopia glasses in other studies.^41^

Women's empowerment was shaped by their social, economic, and political engagements within their communities.^23^ Strong social networks, trust, and support from community members emerged as critical elements, forming a resource often referred to as social capital. Social capital embodies shared identity, trust, and a sense of community, all of which are essential to fostering psychological empowerment.^18^^,^^51^

## 5 Conclusion

This study found that wearing glasses significantly enhanced women's well-being by fostering psychological empowerment (PE) through improved daily life experiences. Using a qualitative phenomenological approach, this research is the first to explore PE through presbyopia correction among older women from the perspectives of both women and men. After presbyopia correction, women reported greater independence—both physically and financially—along with increased confidence, social engagement, and efficiency in their crafts. They also experienced increased income and financial empowerment, enabling them to contribute to household expenses, save money, and make independent financial decisions, with men in the community noting similar benefits to family and community dynamics. Improved vision allowed women to participate more actively in social and political activities, further enhancing their roles in the community. Men recognized the importance of fostering women's autonomy, access to education, and economic participation, emphasizing that these efforts benefit not only women but society as a whole. Supportive family and community relationships played a vital role in reinforcing these experiences, ultimately contributing to greater life satisfaction as women recognized their strengths and challenges within their households and communities.

## Funding

This work was supported by the Excellence in Ophthalmology and Vision Award, 10.13039/100004336Novartis. Basel, Switzerland (grant number NPO 6240 R8898CPH). VFC is supported by 10.13039/100010269Wellcome Trust (grant number R2806CPH).

## Contributorship statement

CG, OJO, AA: Conceptualisation, investigation, methodology, validation and writing – review and editing; RG, EM: Conceptualisation, investigation, methodology, validation and writing – review and editing; FO, VFC: Conceptualisation, data curation, formal analysis, funding acquisition, investigation, methodology, project administration, visualisation, writing - original draft preparation and writing – review and editing.

## Data availability statement

All data relevant to the study has been included in the manuscript and supplemental materials.

## Declaration of competing interest

All author reported no conflict of interests.
